# Current News

**Published:** 1904-03

**Authors:** 


### Current News.

### BILL REGULATING DENTAL PRACTICE IN THE DISTRICT OF COLUMBIA.

[Through the courtesy of Hon. William H. Wiley, of New Jersey, and Hon. A. A. Wiley, of Alabama, we are enabled to lay before the readers of the International the passage of an amendment that may have wide-spread influence upon State legislation in this direction. The bill as passed finally and approved by the President is herewith subjoined.—Ed.]

An Act To amend an Act entitled "An Act for the regulation of the practice of dentistry in the District of Columbia, and for the protection of the people from empiricism in relation thereto," approved June sixth, eighteen hundred and ninety-two.

*Be it enacted by the Senate and House of Representatives of the United States of America in Congress assembled,* That the Act of Congress entitled "An Act for the regulation of the practice of dentistry in the District of Columbia, and for the protection of the people from empiricism in relation thereto," approved June sixth, eighteen hundred and ninety-two, be, and the same is hereby, amended by striking out all of the proviso in section three of said Act and inserting in lieu thereof the following: "*Provided,* That the board of dental examiners may issue a license to practice to any dentist who shall have been in legal practice for a period of five years or more, upon the certificate of the board of dental examiners of the State or Territory in which he practised, certifying his competency and moral character, and upon the payment of the certification fee without examination as to his qualifications."

Approved, February 5, 1904.

---

### MINNESOTA STATE DENTAL ASSOCIATION.

The twenty-first annual meeting of the Minnesota State Dental Association will be held in St. Paul, June 16, 17, and 18, 1904.

Geo. S. Todd,
*Secretary.*

### NATIONAL ASSOCIATION OF DENTAL EXAMINERS.

The National Association of Dental Examiners will hold their annual meeting in the Coliseum building, corner Thirteenth and Olive Streets, St. Louis, Mo., on the 25th, 26th, and 27th of August, beginning promptly at ten A.M. Telephone and telegraph offices in the building. Hotel accommodations will be secured for the members.

Chas. A. Meeker,
*Secretary and Treasurer.*

---

### NEW JERSEY STATE DENTAL SOCIETY.

The New Jersey State Dental Society will hold its annual convention in the Auditorium at Asbury Park, July 21, 22, and 23 next. The Exhibit Committee are prepared to allot space to exhibitors. Make application to the Chairman,

Dr. W. G. Chase.

---

### MINNESOTA STATE BOARD OF DENTAL EXAMINERS.

The Minnesota State Board of Dental Examiners will meet, for the purpose of examining applicants for license, April 5, 6, and 7, 1904. No application received after twelve M., April 5.

Meeting held at Dental Department of State University at Minneapolis.

C. H. Robinson,
*Secretary and Treasurer.*

---

### AMERICAN DENTAL SOCIETY OF EUROPE.

The next annual meeting of the American Dental Society of Europe will be held at the Hamburger Hof, Hamburg, Germany, April 1 to 4, 1904.

Dr. Charles J. Monk,
*Hon. Secretary.*

### DENTAL BOARD OF NEW SOUTH WALES.

I, THE undersigned, hereby notify that the following persons were the successful candidates at the recent election of the Dental Board of New South Wales, held on the eighteenth day of December, 1903:

*Medical Practitioners.*—Sir James Graham, K.B., M.D.; Arthur Palmer, M.B., F.R.C.S.

*Dentists.*—Henry Peach, D.D.S., Cornelius Charles Marshall, Charles Hall, Charles George Hodgson.

And I hereby declare the said Sir James Graham, Arthur Palmer, Henry Peach, Cornelius Charles Marshall, Charles Hall, and Charles George Hodgson to be duly elected as members of the Dental Board of New South Wales.

Witness my hand at Sydney this nineteenth day of December, 1903.

HORACE TAYLOR, J.P.,
*Returning Officer.*

---

### IOWA STATE DENTAL SOCIETY.

THE forty-second annual meeting of the Iowa State Dental Society will be held at Des Moines, Tuesday, Wednesday, and Thursday, May 3, 4, and 5, 1904.

W. R. CLACK, *President,*
Clear Lake, Iowa.
C. W. BRUNNER, *Secretary,*
Toledo, Iowa.

---

### FRATERNAL DENTAL SOCIETY OF ST. LOUIS.

The Fraternal Dental Society of St. Louis meets every third Tuesday evening, at eight o'clock, at the Lindell Hotel. Following are the officers for 1904:

President, Edward Everett Haverstick; Vice-President, W. E. Brown; Secretary, E. P. Dameron; Treasurer, Sam. T. Bassett.

*Executive Committee.*—B. L. Thorpe, W. L. Whipple, Geo. A. Mathae.

### READING DENTAL SOCIETY.

A meeting of the Reading Dental Society was held February 4, 1904. Dr. Walter H. Neall was the guest of honor, and read a paper on "Food and Health from a Dental Stand-Point."

The following officers were elected: President, W. H. Scholl; Vice-President, Charles Grim; Treasurer, Elwood Tate; Secretary, C. R. Scholl.

*Executive Committee.*—George Schlegal, E. M. Bohn, L. E. Tate.

C. R. Scholl,
*Secretary.*

---

### SOUTHERN DENTAL SOCIETY OF NEW JERSEY.

At the annual meeting of the Southern Dental Society of New Jersey, held January 20, 1904, in Camden, the following officers were elected:

President, Alphonso Irwin; Vice-President, W. A. Jacquette; Treasurer, Mary A. Morrison; Recording Secretary, Stanley Ironside; Corresponding Secretary, C. Ironside; Librarian, J. G. Halsey.

*Executive Committee.*—W. W. Crate, J. G. Halsey, Charles P. Tuttle, E. E. Brown, O. E. Peck.

*Membership Committee.*—Alphonso Irwin, W. H. Gelston, W. A. Jacquette.

W. W. Crate,
*Chairman Executive Committee.*

---

### NEW HAVEN DENTAL ASSOCIATION OF CONNECTICUT.

The annual convention of the New Haven Dental Association will be held March 15 and 16, at Harmonie Hall, New Haven, Conn.

This promises to be the largest meeting ever held in the East, a large number of clinics and one of the finest exhibits ever shown.

Essays by the following distinguished members of the dental and medical profession: Drs. R. Ottolengui, New York; Henry C. Boenning, Philadelphia; G. Lenox Curtiss, New York; Herbert L. Wheeler, New York; R. A. McDonnell and Wm. H. Metcalf, New Haven; J. Wesley Shaw, Springfield. A large clinic from residents of New York, New Jersey, Philadelphia, Massachusetts, and Connecticut, and two surgical clinics, provided suitable cases are presented. The business meeting will be dispensed with, thereby allowing ample time to the thorough discussion of all papers.

It will amply repay all to attend this convention, and enable those who have never had an opportunity to visit the Elm City, with the privilege of the freedom of Yale University and the campus.

The Arrangement Committee have been especially active, having arranged for a banquet on the evening of the first day, and in addition are arranging with the Northeast Passenger Association for a one and a third rate on the certificate plan, provided one hundred tickets are sold for seventy-five cents or over. (Secure certificate when purchasing ticket.)

An invitation is extended to all ethical practitioners to join with us, and take active part in our meeting.

FREDERICK H. BROWN, *President.*
E. FRANK COREY, *Secretary.*

NEW HAVEN CONN., February 8, 1904.

---

### PENNSYLVANIA ASSOCIATION OF DENTAL SURGEONS.

THE fifty-seventh annual meeting of the Pennsylvania Association of Dental Surgeons was held at the Continental Hotel, Philadelphia, on Tuesday evening, October 10, 1903. After the reading and discussion of papers the following officers were elected for the ensuing year: President, Wilbur F. Litch; Vice-President, M. I. Schamberg; Secretary, J. Clarence Salvas; Treasurer and Librarian, Wm. H. Trueman.

J. CLARENCE SALVAS,
*Secretary.*

